# Feasibility of a Comprehensive eCoach to Support Patients Undergoing Colorectal Surgery: Longitudinal Observational Study

**DOI:** 10.2196/67425

**Published:** 2025-02-25

**Authors:** A Daniëlle Talen, Jobbe P L Leenen, Geert van der Sluis, Hilbrand K E Oldenhuis, Joost M Klaase, Gijsbert A Patijn

**Affiliations:** 1 Research Group Healthy Ageing, Allied Health Care and Nursing, Groningen Hanze University of Applied Sciences Groningen Groningen The Netherlands; 2 Connected Care Center Isala Hospital Zwolle The Netherlands; 3 Research Group IT Innovations in Healthcare Windesheim University of Applied Sciences Zwolle The Netherlands; 4 Department of Health Innovation Nij Smellinghe Hospital Drachten The Netherlands; 5 Research Group Digital Transformation Hanze University of Applied Sciences Groningen The Netherlands; 6 Department of Surgery University Medical Center Groningen Groningen The Netherlands; 7 Department of Surgery Isala Hospital Zwolle The Netherlands

**Keywords:** eCoach, telehealth, remote monitoring, home monitoring, virtual, eHealth, colorectal surgery, colorectal cancer, prehabilitation, ERAS, rehabilitation, care pathway, patient journey, feasibility, coaching, mobile phone

## Abstract

**Background:**

The mainstay of colorectal cancer care is surgical resection, which carries a significant risk of complications. Efforts to improve outcomes have recently focused on intensive multimodal prehabilitation programs to better prepare patients for surgery, which make the perioperative process even more complex and demanding for patients. Digital applications (eCoaches) seem promising tools to guide patients during their care journey. We developed a comprehensive eCoach to support, guide, and monitor patients undergoing elective colorectal surgery through the perioperative phase of the care pathway.

**Objective:**

The primary aim of this study was to determine its feasibility, in terms of recruitment rate, retention rate, and compliance. Also, usability and patient experience were examined.

**Methods:**

A single-center cohort study was conducted from April to September 2023 in a tertiary teaching hospital in the Netherlands. All elective colorectal surgery patients were offered an eCoach that provided preoperative coaching of the prehabilitation protocol, guidance by giving timely information, and remote monitoring of postoperative recovery and complications. Recruitment and retention rate, as well as compliance for each part of the care pathway, were determined. Secondary, patient-reported usability measured by the Usefulness, Satisfaction, and Ease of Use questionnaire and patient experiences were reported.

**Results:**

The recruitment rate for the eCoach was 74% (49/66). Main reasons for exclusion were digital illiteracy (n=10), not owning a smartphone (n=3), and the expected burden of use being too high (n=2). The retention rate was 80% (37/46). Median preoperative compliance with required actions in the app was 92% (IQR 87-95), and postoperative compliance was 100% (IQR 100-100). Patient-reported usability was good and patient experiences were mostly positive, although several suggestions for improvement were reported.

**Conclusions:**

Our results demonstrate the feasibility of a comprehensive eCoach for guiding and monitoring patients undergoing colorectal surgery encompassing the entire perioperative pathway, including prehabilitation and postdischarge monitoring. Compliance was excellent for all phases of the care pathway and recruitment and retention rates were comparable with rates reported in the literature. The study findings provide valuable insights for the further development of the eCoach and highlight the potential of digital health applications in perioperative support.

## Introduction

### Background

Colorectal cancer is the third leading cause of cancer deaths worldwide and is mainly diagnosed at an advanced age [1]. The mainstay of colorectal cancer care is surgical resection, which carries a significant risk of complications [2,3]. During hospital admission, enhanced recovery after surgery programs have been adopted widely, resulting in shorter hospital lengths of stay [4,5]. More recently, focus has shifted to optimizing patients preoperatively through multimodal prehabilitation programs, including physical training programs, improving nutritional status, and ameliorating medical comorbidities, thereby reducing postoperative complications [6,7]. After discharge, patients are encouraged to actively rehabilitate to full functional recovery. The entire care pathway from diagnosis to full functional recovery generally takes several months or longer when patients need to receive (neo)adjuvant chemo(radiation) therapy.

For many patients, the perioperative journey can be overwhelming and increasingly complex, as they need to manage a lot of information and perform various tasks at different times [8]. This highlights the need for a broader approach to health care that focuses not just on treating the disease but also on overall well-being, long-term recovery, and self-management. To support patients better, digital tools such as eCoaches are being used more in clinical practice [9]. These tools offer timely information, reminders, and remote monitoring to help patients stay on track and detect complications early. By promoting self-management, eCoaches also reduce the burden on health care systems, which is crucial as resources become more limited [10].

Many health apps for perioperative guidance are available, but the content is often narrow and applied to only one aspect of the care pathway, such as prehabilitation or postoperative monitoring [11-14]. An eCoach for colorectal surgery was reported, but did not include prehabilitation, for which digital coaching can be particularly helpful [15]. Furthermore, clear reporting of feasibility for older surgical patients in real-life clinical practice is often missing [16,17]. A recent study described feasibility of an intervention that combined digital guidance with intensive one-on-one human health coaching, but this is a health professional labor-intensive protocol [18]. More comprehensive digital coaching applications are needed that minimize health care resource usage while optimally informing and engaging patients, ultimately enhancing the quality of care.

A comprehensive eCoach was implemented to guide the patient throughout the perioperative colorectal pathway, providing timely information and monitoring prehabilitation adherence. In addition, immediately after discharge, patients were monitored remotely (vital signs, vomiting, stools, pain, and wound healing with automated alert identification and handling) to allow early detection of postoperative complications and thereby potentially prevent emergency readmissions and improve outcomes. To our knowledge this is the first eCoach for elective colorectal surgery encompassing the entire care pathway, including prehabilitation as well as postdischarge postoperative monitoring. This study explores the feasibility of a digital health application by assessing whether it works as intended in a given context, emphasizing key factors for implementation success while also considering user experience and system demands.

### Aim

The primary aim of this study was to determine the feasibility, in terms of recruitment rate, retention rate, and compliance, of a comprehensive eCoach in support of the perioperative care pathway for colorectal surgical patients. The secondary aim was exploring usability, patient experiences and feedback, and evaluating app-induced workload.

## Methods

### Study Design and Setting

A single-center longitudinal observational study was conducted from April 2023 until September 2023 in a 1200-bed tertiary teaching hospital (Isala, Zwolle) in the Netherlands. Annually, approximately 350 colorectal resections are performed by a team of 5 specialized general surgeons. In April 2023, the eCoach application was implemented into the colorectal surgery pathway at the same time as the implementation of a standardized multimodal prehabilitation program (Fit4Surgery [19]). The STROBE (STrengthening the Reporting of OBservational studies in Epidemiology) guideline for reporting observational studies was followed [20].

### Ethical Considerations

The Medical Ethics Committee of the Isala Hospital reviewed the protocol (20230403) and declared that the Medical Research Involving Human Subjects Act (also known by its Dutch abbreviation WMO) did not apply for this study, as the study involves an evaluation of usual care data. The study was conducted in accordance with the Declaration of Helsinki. After onboarding in the eCoach, each patient provided informed consent for use of their personal health information for research purposes in the app.

### Participants and Procedures

Patients (older than 18 years) who were preparing for elective colorectal surgery and following the prehabilitation program were included when they were able to communicate in Dutch. The application was integrated into “usual care,” whereby the surgeon explicitly advised patients during their preoperative visit to enroll in the prehabilitation program and to use the app. The nurse coordinator checked for eligibility right after the appointment with the surgeon by asking, “do you have a smartphone?” and “are you good at using your smartphone?” Patients were excluded if they were unable to use the app because they did not own a smartphone, did not have web connection, did not possess sufficient digital literacy skills, or had preexistent physical or mental limitations. The onboarding process was completed during an appointment with the case manager (a specially trained nurse who performs the screening and coordination of prehabilitation), who explained the use of the eCoach and evaluated the patient’s ability to use it effectively. Patients who underwent emergency surgery during the care pathway, prior to the planned colorectal resection (eg, due to bowel obstruction), were excluded from the study. When patients received neoadjuvant treatment (radiotherapy or chemoradiotherapy), they were included only after completion and restaging (response evaluation) and definitive acceptance for surgery by the colorectal multidisciplinary team meeting. Health care professionals registered in the electronic patient files if patients were eligible, reasons for nonparticipation, and all usual care data.

### Intervention Description

The mobile app eCoach (Luscii Healthtech BV) was developed by health care professionals (clinicians, physiotherapists, dieticians, and nurse practitioners) with expertise on perioperative care in collaboration with the Isala Connected Care team and Luscii Healthtech BV (Multimedia Appendix 1).

**Figure 1:**
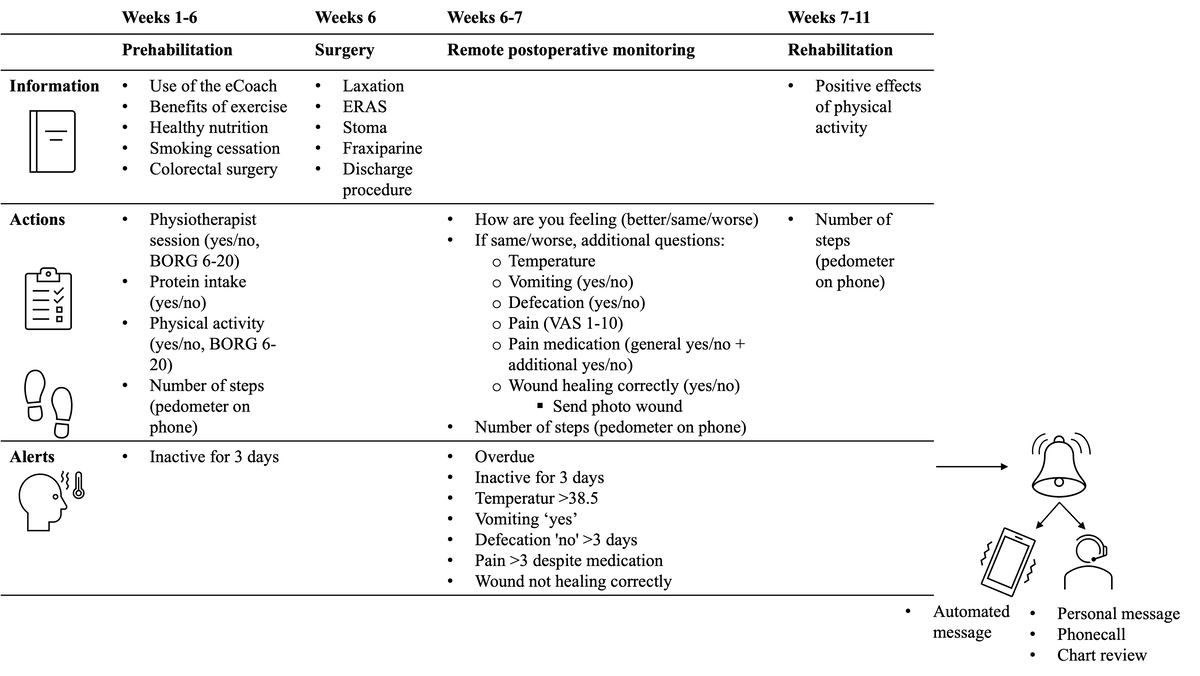
Overview of the eCoach intervention in the colorectal care pathway. BORG: Borg Rating of Perceived Exertion Scale; ERAS: enhanced recovery after surgery protocol; VAS: visual analogue scale.

Figure 1 illustrates the perioperative care pathway and the integration of the eCoach into this process, including the phases of prehabilitation, surgery, remote postoperative monitoring, and rehabilitation. The eCoach provided tailored information and action prompts specific to each phase. Automated alerts were configured and managed by specialized virtual care nurses at the Isala Virtual Care Center. If required actions were not completed, an automated reminder was sent in the evening. Inactivity for more than 3 consecutive days triggered an alert to the virtual care nurse, who could then take appropriate action, such as sending a personal message, making a phone call, or reviewing the patient’s chart and deciding that no action was necessary. The eCoach acts as a gatekeeper, with all processes being highly standardized and objective. The virtual care nurse reviews the situation when an alert is triggered and determines the appropriate action based on the specific circumstances. This ensures that patient management is consistent and reliable, while allowing for personalized intervention when necessary.

In the prehabilitation phase (weeks 1-6), the eCoach monitored adherence to the multimodal prehabilitation program and provided timely information on relevant aspects of the care pathway. This involved prompting patients to report whether they attended a physiotherapy session that day and to record their Borg Rating of Perceived Exertion (BORG 6-20) as soon as possible afterward. To support adherence to the nutritional component, the eCoach inquired whether patients had taken their prescribed protein supplement.

During the surgery phase (week 6), the eCoach provided information on key topics such as preparing for surgery, bowel preparation (if applicable), anticoagulant therapy, and discharge procedures.

In the remote postoperative monitoring phase (weeks 6-7), patients completed a daily questionnaire assessing how they felt compared with the previous day (better, the same, or worse). If they reported feeling the same or worse, the eCoach prompted additional questions about body temperature, pain, vomiting, defecation, and wound healing. If responses exceeded set thresholds, an alert was generated, and an automated message advised the patient to contact the hospital. The virtual care nurse checked the alerts and took action when necessary. This included validating the alerts (eg, requesting wound details and photographs and forwarding them to the responsible department) and ensuring that patients followed the app’s advice to contact the hospital.

In the rehabilitation phase (weeks 7-11) the eCoach provided information about the positive effect of physical activity in recovery after surgery and monitoring of physical activity. Although the care pathway transitions into long-term cancer follow-up, in this feasibility study, it was considered to end at 30 days post surgery.

### Variables and Measurements

#### Primary Outcomes

The primary outcome of this study was the feasibility of the eCoach app. Feasibility explores whether a digital health system works as intended in a given context and was measured by the recruitment rate, retention rate, and compliance [21]. Recruitment rate was calculated as the proportion of eligible patients, relative to the total elective colorectal surgery patient cohort during the study period. Retention rate was defined as the proportion of patients who completed the use of the eCoach until the end of the eCoach care pathway (30 days after surgery), with reasons for dropout documented. Compliance was defined as the extent at which patients followed the prescribed actions (as shown in Figure 1) within the app, as presented in the intervention description. Since the rates reported in previous literature range between 53% and 95%, we deemed the eCoach feasible when the recruitment rate, retention rate, and compliance were all above 70% [17].

#### Secondary Outcomes

The secondary outcomes were patient experiences (usability and feedback), app-induced nursing activities, and preliminary effectiveness parameters. A full description of the operationalization of the secondary outcomes can be found in Multimedia Appendix 2.

Usability was evaluated using the Usefulness, Satisfaction, and Ease of Use (USE) questionnaire, which consists of 30 statements rated on a 7-point Likert scale [22]. These statements pertain to 4 key constructs: usefulness, satisfaction, ease of use, and ease of learning regarding the interventions. The questionnaire had been translated into Dutch and used in prior research, with Cronbach a per construct from 0.916 to 0.965 [23,24]. It was gathered using an automatic message in the eCoach, which included a link to the questionnaire.

Feedback on the app’s use was collected at the end of the telemonitoring process by the virtual care nurse through a phone call, which was documented in the electronical patient dossier. During the call, patients were asked open-ended questions such as, “How did you experience this process?” and “What improvements would you suggest?” Patient feedback was coded and the themes were categorized into “positive experiences” and “proposed improvements” applying the principles of content analysis [25]. Coding and thematizing was performed by 2 researchers (ADT and JPLL) who discussed differences until consensus was reached. The number of times a theme was mentioned by a patient was reported.

App-induced nursing activities were determined by describing the number of alerts per action item as described in Figure 1 and type of nurse actions that were initiated by alerts of the eCoach.

Preliminary effectiveness parameters consisted of preoperative outcomes after prehabilitation (Steep Ramp Test, 1 repetition maximum tests, and Patient-Generated Subjective Global Assessment Short Form), perioperative functional outcomes (quality of recovery, physical functioning, and quality of life), and postoperative parameters (postoperative complications, length of stay, and time to functional recovery). The Quality of Recovery-15 (QoR-15), Patient-Reported Outcomes Measurement Information System (PROMIS)–Physical Function (PF), and PROMIS-10 questionnaires were administered preoperatively (1 day before surgery) and postoperatively (2 days, 7 days, and 30 days) automatically in the eCoach app. The virtual care nurse made a scheduled call to all patients to remind them about the 30-day questionnaires and asked their feedback on the process. Patient characteristics and postoperative parameters of the study population were gathered (Table 1).

**Table 1:** Patients’ characteristics.

Characteristics		Study population (n=37)
Sex (female), n (%)		17 (46)
Age (year), median (IQR)		65 (60-77)
BMI, median (IQR)		26 (24-32)
**ASA^a^, n (%)**		
	I	3 (8)
	II	24 (65)
	III	9 (24)
	IV	1 (3)
CCI^b^, median (IQR)		5 (4-6)
Type of surgery (laparoscopic), n (%)		36 (97)
**Tumor location, n (%)**		
	Colon	27 (73)
	Rectum	10 (27)
Tumor sort (malignant), n (%)		36 (97)
**Surgery procedure, n (%)**		
	Right hemicolectomy	14 (38)
	Left hemicolectomy	5 (14)
	Sigmoid resection	8 (22)
	LAR^c^	7 (19)
	APR^d^	2 (5)
	Stoma	1 (3)
Smoking (yes), n (%)		4 (11)
VSAQ^e^, median (IQR)		8 (6-10)
HADS^f^, median (IQR)		5 (3-8.5)
**Hemoglobin (mmol), median (IQR)**		
	Baseline (n=33)	8.2 (7.0-9.2)
	Preoperative (n=17)	7.6 (6.0-9.1)
Complications (yes), n (%)		8 (22)
**Clavien-Dindo, n (%)**		
	I-II	4 (11)
	III	3 (8)
	IV	1 (3)
Length of stay in days, median (IQR)		4 (3-5)
Time to functional recovery, median (IQR)		1 (0-2)
**Care after discharge, n (%)**		
	Independent	28 (76)
	Home care	9 (24)
	Rehabilitation center	N/A^g^
Readmissions (yes), n (%)		3 (8)
SRT^h^ (W^i^/kg), median (IQR)		2.61 (2.10-3.68) before prehabilitation^j^; 2.82 (2.16-4.10) after prehabilitation^k^
**1 RM^l^ tests, median (IQR)**		
	Row	30 (24-35) before prehabilitation; 35 (28-43) after prehabilitation
	Chest press	40 (26-56) before prehabilitation; 53 (35-60) after prehabilitation
	Leg press	210 (183-323) before prehabilitation; 235 (188-299) after prehabilitation
	Lat pulldown	32 (26-38) before prehabilitation; 35 (29-42) after prehabilitation
PG-SGA sf^m^, median (IQR)		1 (0.5-4) before prehabilitation; 1 (0-2) after prehabilitation

^a^ASA: American Society of Anesthesiologists.

^b^CCI: Charlson Comorbidity Index.

^c^LAR: low anterior resection.

^d^APR: abdominal perineal resection.

^e^VSAQ: Veteran Specific Activity Questionnaire.

^f^HADS: Hospital Anxiety and Depression Scale.

^g^N/A: not applicable.

^h^SRT: Steep Ramp Test.

^i^W: wattage.

^j^Before prehabilitation: n=34.

^k^After prehabilitation: n=26.

^l^RM: repetition maximum.

^m^PG-SGA sf: Patient-Generated Subjective Global Assessment Short Form.

### Statistical Analysis

Formal sample size calculation was challenging given the observational feasibility study design, but a sample in the range of 20-25 is considered adequate for this type of study [26,27]. We determined to include all patients during 3 months. Given the expected number of surgeries (n=85) and dropout rate of previous studies (50%), we expected to include 40 patients.

Descriptive statistics were used to evaluate patient demographics and to assess the feasibility. Continuous data were checked for normality by a Shapiro-Wilk test and visually by a histogram. Based on normality, median and IQR or mean and SD were presented. For categorical data, frequencies and percentages were calculated. All data were analyzed using SPSS Statistics (version 24; IBM Corp) for Windows. The answers on the open-ended questions were coded and categorized by 2 researchers (ADT and JPLL) by content analysis based with predefined categories: positive experiences and proposed improvements. Categories were quantified.

## Results

### Overview

A total of 37 patients completed the study, of which the patient characteristics are shown in Table 1. Median age was 65 (IQR 60-77) years and median Charlson Comorbidity Index score was 5 (IQR 4-6). Four patients experienced minor complications (Clavien Dindo I-II), such as issues with ileostomy production and atrial fibrillation. The other 4 patients had major complications (Clavien Dindo III-IV), including abscess, anastomotic leakage, and systemic inflammatory response syndrome, with 1 patient requiring intensive care unit admission.

### Primary Outcomes

#### Recruitment Rate and Retention Rate

**Figure 2:**
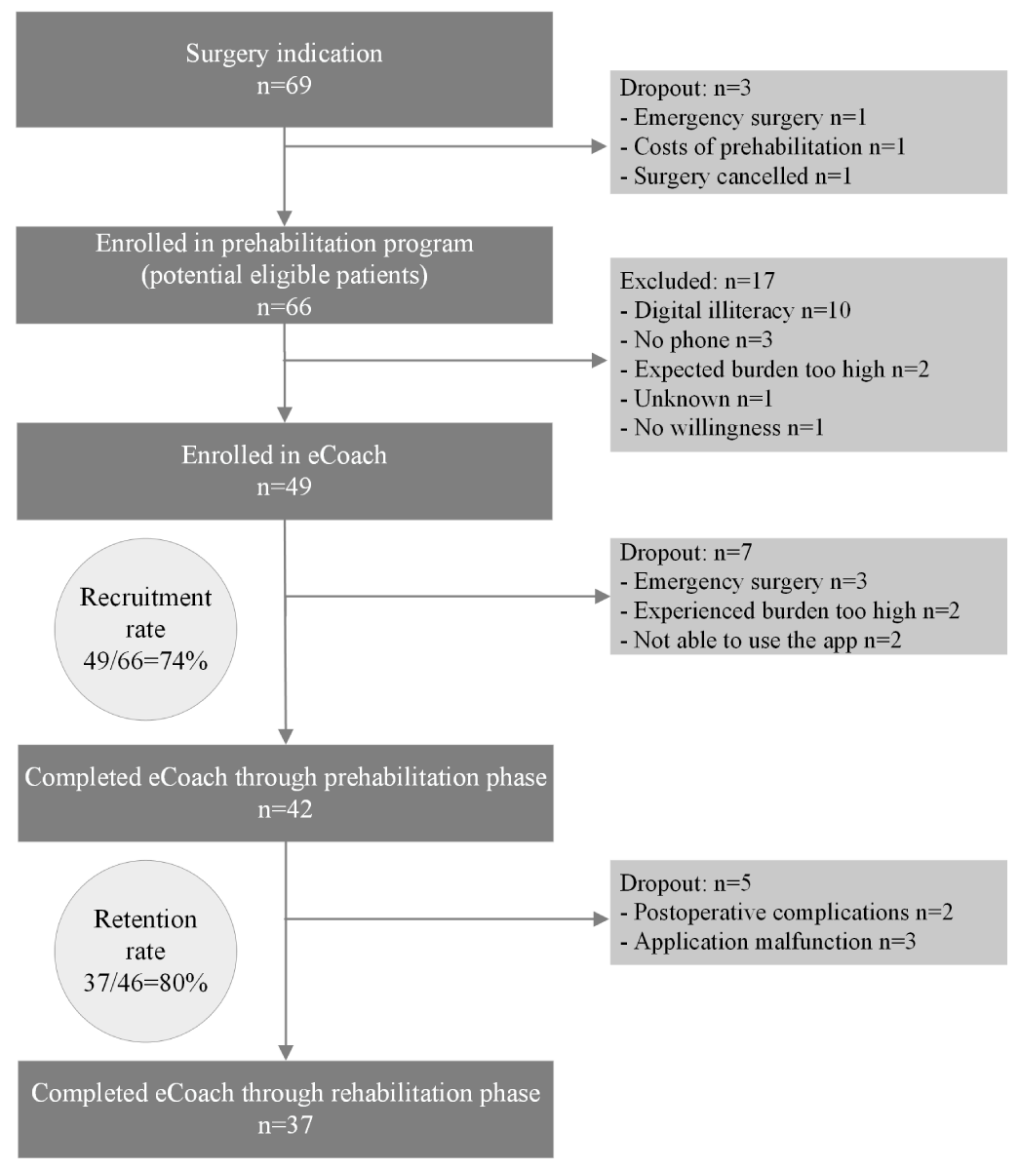
Flowchart of study population, including recruitment rate and retention rate.

During the study period 66 patients were eligible for the colorectal surgery pathway. Of the 66 included patients, 49 enrolled in the eCoach, resulting in a recruitment rate of 74% (49/66; Figure 2). Main reasons for exclusion were digital illiteracy (n=10), not having a phone (n=3), and expected extra burden of the app being too high (n=2). Of the 49 enrolled patients, 4 (92%) dropped out preoperatively during the prehabilitation phase and 5 (88%) postoperatively during the close monitoring or rehabilitation phase, resulting in a retention rate of 80%. Two patients dropped out due to the significant burden imposed by postoperative complications, leading them to discontinue using the eCoach. The 3 patients who underwent emergency surgery were excluded from the calculations of recruitment and retention rates.

#### Compliance

Median compliance was 95% (IQR 82%-96%), preoperative compliance was 92% (IQR 87%-95%), and postoperative compliance was 100% (IQR 100%-100%). Preoperative compliance was highest with 98% (IQR 90%-100%) with “physiotherapist session.” Compliance with the number of steps was lowest with 86% (IQR 72%-93%). Compliance with “protein intake” was 90% (IQR 84%-97%), on which patients reported “yes” 97% of the time. Of the 37 patients, 34 patients responded. The median compliance to the postoperative questions “Well-being compared to yesterday” was 100% (IQR 100%-100%), and patients reported feeling better 60% of the times. Compliance for the additional questions was 100% (IQR 100%-100%), where “Wound healing correctly” resulted in a negative response most of the times. A comprehensive presentation of compliance with various components of the eCoach is shown in Multimedia Appendix 3.

### Secondary Outcomes

#### Patient Experiences: Usability

**Figure 3:**
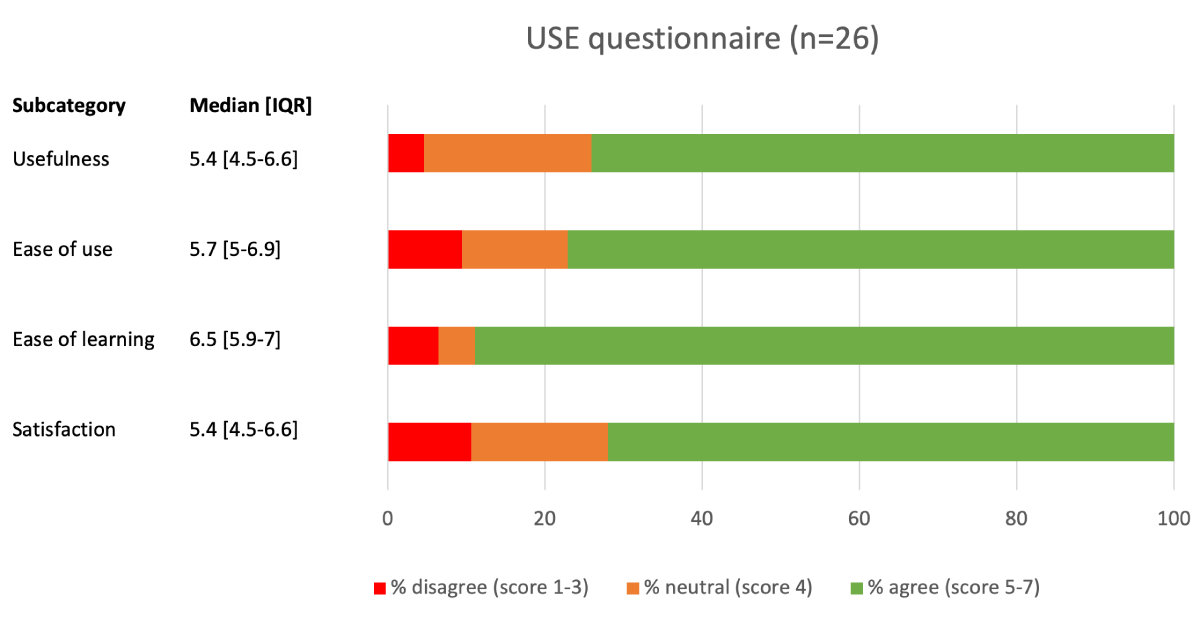
Usefulness, Satisfaction, and Ease of Use questionnaire. USE: Usefulness, Satisfaction, and Ease of Use.

Twenty-six patients (response rate: 70%) completed the USE questionnaire (Figure 3) at day 30 postoperatively. Median scores for usefulness, ease of use, ease of learning, and satisfaction were 5.4, 5.7, 6.5, and 5.4, respectively, on a 1-7 Likert scale, all of which are considered good outcomes. Scores and IQRs to individual questions and categories are described in detail in Multimedia Appendix 4.

#### Patient Experiences: Content Analysis

In total, 89% (33/37) of patients answered the questions about their experiences with the eCoach. Forty-eight positive experiences were reported. One patient said, “I especially valued the motivation to stay physically active. I feel like this made me healthier and stronger.” Other patients called it “a good incentive,” or “a helpful reminder for the protein intake.” Twenty-four patients reported 41 proposed improvements. These areas of improvement were diverse, but rigidity of the app was most frequently mentioned (Textbox 1 and Multimedia Appendix 5).

**Content analysis of experiences reported by patients.**

**Positive experiences (n=48)**

- General positive experiences (n=18)
- Providing support and engagement (n=15)
- Informative (n=7)
- Stimulating motivation and incentives (n=4)
- Mental support (n=2)
- Continuous connection (n=2)

**Proposed improvements (n=41)**

- Limited usability (n=9)
- Rigidity of the app (n=14)
- Problems with the pedometer (n=6)
- Length of postoperative monitoring was unclear or insufficient (n=6)
- Missed features in the app (n=3)
- Engagement difficulties and mental burden (n=3)

#### App-Induced Nursing Activities

Out of 1752 preoperative alerts, 99.9% (n=35) were processed automatically by the eCoach, with only 2 alerts needing manual interventions for protein intake. Of the 222 alerts for protein intake across 30 patients, 99% (n=29) were processed automatically by the system. Two alerts required manual interference, so 2 messages were sent by the nurse to remind patients about their protein intake. The number of postoperative alerts was 126 (n=10), of which 43% (54/126) were processed automatically. The remaining alerts led to 21 phone calls and 19 messages in the app. The alerts for “Wound healing correctly” resulted in the most alerts with actions necessary. A detailed summary of nursing activities induced by the app is shown in Multimedia Appendix 6.

#### Preliminary Effectiveness Parameters

The median physical fitness was preoperatively 2.61 (IQR 2.10-3.68) W/kg and postoperatively 2.82 (IQR 2.16-4.10) W/kg on the Steep Ramp Test (Table 1). Eight patients developed complications after surgery (8/37, 22%) and 4 of them severe (Clavien Dindo III or IV) (4/37, 11%). Median length of stay was 4 days (IQR 3-5) and median time to functional recovery was 1 day (IQR 0-2).

Quality of recovery at 30 days postsurgery was rated comparable with preoperative scores, whereas quality of life and physical functioning at 30 days were not completed back to preoperative levels (Table 2).

**Table 2:** Preliminary effectiveness parameters.

	–2 days (n=22)	+1 day (n=23)	+7 days (n=24)	+30 days (n=26)
Quality of recovery score, median (IQR)	134.5 (105-144)	112.0 (83-120)	119.5 (102-133)	135 (115-143)
PROMIS^a^-10 (quality of life) score, median (IQR)	28 (25-34)	26 (23-29)	N/A^b^	26 (25-32)
PROMIS of Physical Functioning score, median (IQR)	38.5 (23.8-40)	13 (11-16)	N/A	28.5 (20-34)

^a^PROMIS: Patient-Reported Outcomes Measurement Information System.

^b^N/A: not applicable.

## Discussion

### Principal Findings

Our results demonstrate the feasibility of a comprehensive eCoach that was developed for elective colorectal surgery patients incorporating all phases of the care pathway, including prehabilitation, enhanced recovery after surgery components, and postoperative monitoring. Recruitment (49/66, 74%) and retention (37/46, 80%) rates were comparable with rates reported in the literature, whereas the compliance (overall 95%) was excellent. Patient-reported usability was good, and patients not only reported to value the eCoach as a beneficial addition to the patient journey but also reported some areas of improvement that need to be addressed in future iterations of the eCoach.

### Recruitment and Retention

We found that a significant number of eligible patients were unable to use the eCoach. Nineteen patients (19/66, 29%) were excluded at baseline due to digital illiteracy, not owning a smartphone, or finding the eCoach mentally burdensome. Only 1 patient was excluded due to unwillingness to participate. Given the study population of unselected patients with colorectal cancer, including a significant proportion of older adult and frail patients, this finding is, however, not unexpected and in line with recruitment rates reported in the literature [11,18,28-31]. Older age groups are known to have lower digital proficiency and lower smartphone ownership, and using a digital application may provide a high perceived burden for frail older adult patients, who are facing the challenges of a recent cancer diagnosis and an upcoming high-risk surgery [17]. Although our study did not quantify frailty, the reasons for nonparticipation, such as digital illiteracy and lack of smartphone ownership, suggest that excluded patients were more likely to be older adults and vulnerable. This aligns with findings from a digital prehabilitation study, which reported that patients with insufficient digital skills were older and had a more unfavorable risk profile [32].

Retention was comparable or slightly better than rates reported in the literature. Dropouts were disease related, technical, or due to a perceived heavy burden of using the eCoach. The reported technical issues (malfunction) had not been encountered during initial beta testing and were promptly solved by the development team. When dropping out in the preoperative phase due to emergency surgery (eg, bowel obstruction), these patients could no longer participate for external reasons and were thus excluded from the retention assessment.

Our findings show that there is a subset of patients unable to participate or dropping out for various reasons, confirmed by previous studies. Thus, in clinical practice we cannot relying solely on digital coaching. Hybrid approaches including nondigital and personalized coaching and guidance will remain necessary in order to reach all patients. Furthermore, these results highlight the need for more inclusive design in health care technology, ensuring that the development process considers vulnerable groups and digital illiteracy.

### Compliance

Overall compliance (adherence) was excellent in our study. It was slightly lower during the preoperative phase (92%) than postoperatively (100%), probably because there were more preoperative actions to comply with. Median compliance for monitoring physiotherapy visits, physical activity, and protein intake varied between 90% and 98%, where in other studies compliance ranged from 53% to 86% [17]. Possible reasons for the high compliance rates are the user-friendly app design, the seamless integration into routine care, the onboarding meeting with a dedicated case manager, and the explicit encouragement to enroll and adhere from the surgeons. Our results underscore the high potential value of digital applications for encouraging patient engagement and self-management, which may help improve quality of care and reduce the number of unplanned patient-provider contacts.

### Usability and Patient Satisfaction

Most of the patients were positive about the guidance by the eCoach, as shown in the high median scores of the USE questionnaire and in the qualitative feedback. Patient feedback has shown that the eCoach also provides implicit psychological support, helping them feel more connected, confident, and mentally prepared for surgery. The qualitative evaluation of a comparable app reported similar responses [33]. Assistance by trained staff is known to increase perceived usability, which might have contributed to our favorable outcomes [17]. Some areas of improvement were reported, including missing features and lack of personalization, which will need to be addressed in future iterations of the eCoach. To further improve patient experiences in real-life practice, the suggested areas of improvement in the feedback should be addressed. Some of these are technical issues, such as links not working correctly, the inability to fill in missed actions a day later, or problems with the pedometer. The problems with the pedometer seem more consistent, since the compliance is structurally lower than the other preoperative actions and the number of alerts, which were all automatically processed, was large. One explanation maybe that some patients did not have a pedometer installed on their smartphone, so often just filled in an estimated number of steps. Furthermore, patients reported inadequate personalization of the eCoach. For instance, patients who were unable to walk (but able to use a bike trainer) felt like the eCoach was not always fitting to their personal situation.

### Strengths and Limitations

The key strength of this study is the comprehensive evaluation of the eCoach in real-life clinical practice, covering the entire perioperative pathway for colorectal surgery patients. This practical implementation allows for efficient assessment of feasibility, addressing both patient needs and implementation challenges early on, with high generalizability within the standardized Dutch health care system. The eCoach platform is commercially available and has been adopted by several centers in the Netherlands. In our center, it has become the standard of care and expanded to include complex surgical patients. To support broader implementation, we are committed to training virtual care nurses, sharing our experiences, and establishing virtual care centers. Ensuring that the necessary infrastructure and expertise are in place is essential, and we are actively working toward this to facilitate the expansion of virtual care services [34].

The results of this observational feasibility study have to be interpreted in the light of some limitations, including its single-center design in which the intervention was accessible only to Dutch-speaking people with a web-connected device, and the relatively small cohort. Another limitation of this study is that we included the first group of patients after implementation of the eCoach, potentially resulting in the technical errors experienced by patients. Although these errors were readily addressed during the initial phase of the study, 3 patients had dropped out as a result. It is important to note that these technical issues were part of the initial learning curve and are expected to be minimized in future iterations of the eCoach, ensuring a smoother experience for subsequent patient groups. Furthermore, more alerts were generated initially as we opted to err on the side of caution to ensure patient safety.

### Future Directions

Further studies in larger cohorts are needed to assess the potential role of an eCoach in improving clinical effectiveness and cost-efficiency, such as its impact on readmission rates and length of stay, by comparing it with a matched historical control group or randomization [34]. eCoaches may help reduce the burden on the health care system by promoting self-management and compliance and thereby reduce the number of unplanned patient-health care provider contacts. The integration of eCoaches into complex care pathways facilitates comprehensive health management, including approaches that extend beyond traditional disease treatment. Combining the eCoach with an objective measurement device, such as an accelerometer or a continuous vital signs monitoring device, may help reliably measure physical activity and assess time to full recovery [35]. The value of the reported additional secondary end points (QoR, PROMIS-10, and PROMIS-PF) measuring preliminary effectiveness and patient-reported functional parameters were of limited value for this study but will be valuable in future follow-up studies.

As recruitment in virtual eCoach applications will remain suboptimal in older adult or frail patients, studies are needed to develop protocols to better triage patients at baseline to select who are eligible and suitable for inclusion. The complexity of health needs and potential cognitive or physical limitations in older adult, frail, and high-risk patients underscore the need for alternative methods of perioperative support. One way to improve recruitment, retention, and intervention efficacy is designing more personalized and tailored digital health applications [36]. Future iterations of the eCoach may facilitate individual exercise mode by personal choice and tailored communication to the individual level of health literacy and education. Adding a web-based interface or the ability to add a caregiver could reduce the technological barrier for some patients.

Current prehabilitation protocols for colorectal surgery include frequent physical training sessions by physiotherapist. Given the excellent compliance of the eCoach, eligible lower-risk patients may well follow an unsupervised virtual prehabilitation program by using the eCoach. A study to determine the value of unsupervised virtual prehabilitation is planned to start at our institution.

### Conclusions

Our results demonstrate the feasibility of using a comprehensive eCoach for guidance and monitoring of elective colorectal surgery patients through all phases of the care pathway. Compliance was excellent and recruitment and retention rates were comparable with rates reported in the literature. Patient-reported usability was good, and patients reported to value the eCoach as a beneficial addition to the patient journey. The study findings provide valuable insights for the further development of the eCoach and highlight the potential of digital health applications in perioperative support.

## Appendix

Multimedia Appendix 1 Examples of the eCoach interface, showing daily actions for patients on the left and a small part of the available information on the right.

Multimedia Appendix 2 Description of secondary outcomes.

Multimedia Appendix 3 Compliance of the different items of the eCoach.

Multimedia Appendix 4 USE outcomes.

Multimedia Appendix 5 Quotes of patient experiences.

Multimedia Appendix 6 App-induced nursing activities.

## Abbreviations

BORG 6-20: Borg Rating of Perceived Exertion
PROMIS: Patient-Reported Outcomes Measurement Information System
QoR-15: Quality of Recovery-15
STROBE: STrengthening the Reporting of OBservational studies in Epidemiology
USE: Usefulness, Satisfaction, and Ease of Use
