# Supplementary figures and images for: Feasibility of a Comprehensive eCoach to Support Patients Undergoing Colorectal Surgery: Longitudinal Observational Study
Source: JMIR Perioper Med. 2025 Feb 25;8:e67425. doi: 10.2196/67425 (PMC11897663; doi:10.2196/67425)

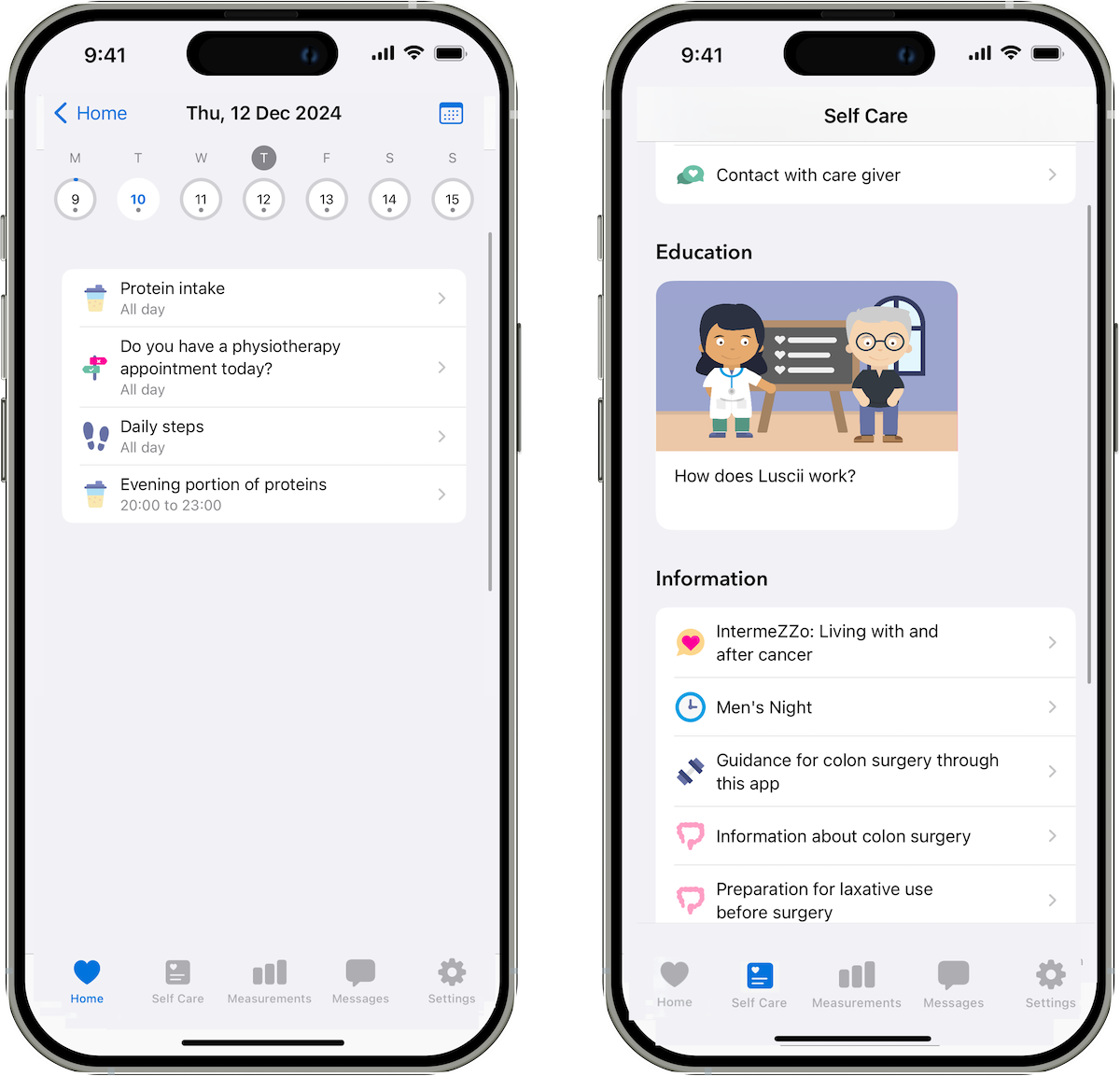

Supplement: Multimedia Appendix 1 [file periop_v8i1e67425_app1.png]
