# Supplementary material for: Feasibility of a Comprehensive eCoach to Support Patients Undergoing Colorectal Surgery: Longitudinal Observational Study
Source: JMIR Perioper Med. 2025 Feb 25;8:e67425. doi: 10.2196/67425 (PMC11897663; doi:10.2196/67425)
Supplement: Multimedia Appendix 2 [file periop_v8i1e67425_app2.docx]

**Multimedia** **Appendix 2. Description of secondary outcomes.**

| Variable | Description |
| --- | --- |
| Age | The age of the patient at the time of enrollment in the study. |
| Gender | The gender of the patient (male/female). |
| BMI | Body Mass Index, calculated as weight in kilograms divided by height in meters squared (kg/m²). |
| ASA | The American Society of Anesthesiologists (ASA) Physical Status Classification System, which assesses the fitness of patients before surgery. Categories range from I (healthy) to IV (severe systemic disease). |
| CCI | Comorbidities assessed using the Charlson Comorbidity Index (CCI), which predicts the ten-year mortality for a patient with a range of comorbid conditions. |
| **Type of Surgery (Laparoscopic)** | Indicates whether the surgery was performed laparoscopically (minimally invasive) or through open surgery. |
| **Tumor Location** | The anatomical location of the tumor, categorized as colon or rectum. |
| **Tumor Type (Malignant)** | Indicates whether the tumor is malignant (cancerous). |
| **Surgery Procedure** | The specific type of surgical procedure performed, such as right hemicolectomy, left hemicolectomy, sigmoid resection, low anterior resection (LAR), abdominoperineal resection (APR), or creation of a stoma. |
| **Smoking Status** | Indicates whether the patient is a smoker (yes/no). |
| **Veterans Specific Activity Questionnaire (VSAQ)** | A tool used to assess functional capacity and predict exercise tolerance. The VSAQ is a tool used to assess functional capacity and predict exercise tolerance. It consists of a 13-point scale where patients indicate the level of activity at which they would need to stop due to limitations. Scores are converted to metabolic equivalents (METs), with higher scores indicating better exercise capacity. For example, a score of 5 on the VSAQ corresponds to activities like walking briskly at 4 mph (6.4 km/h). |
| **Hospital Anxiety and Depression Scale (HADS)** | A 14-item scale used to screen for anxiety and depression, with subscales for each condition. The HADS is a 14-item tool used to screen for anxiety and depression. Each item is rated on a 4-point scale (0-3), with total scores ranging from 0 to 21 for each subscale. Scores of 0-7 are considered normal. Higher scores indicate greater levels of anxiety or depression. |
| **Hemoglobin** | The concentration of hemoglobin in the blood, measured in millimoles per liter (mmol/L). Median and interquartile range (IQR) are reported. |
| Postoperative complications | Complications occurring after surgery, classified according to the Clavien-Dindo classification system. A widely used system for categorizing surgical complications based on their severity, ranging from Grade I (minor complications) to Grade V (death). This classification helps standardize the reporting of surgical outcomes. |
| Length of stay | The number of days from hospital admission to discharge. |
| Time to functional recovery | Time to functional recovery was measured by determining the time in days between surgery and functional recovery, defined as 1) adequate pain control with oral analgesia only, 2) restoration of mobility to an independent or preoperative level, 3) absence of intravenous fluid administration, and 4) ability to eat solid foods. This was reported by the clinical nurses and checked by the first author. |
| Baseline functional status | The initial level of physical functioning of the patient, assessed using standardized physical tests during preoperative assessment (approximately 30 days before surgery) and after following a prehabilitation program. |
| Steep Ramp Test | A physical test to measure the patient's aerobic capacity and endurance. |
| 1 Repetition Maximum Tests | Tests to determine the maximum amount of weight a patient can lift in a single repetition for various exercises on exercise machines, including low row, chest press, leg press and lat pulldown. |
| PG-SGA SF | Patient Generated Subjective Global Assessment Short Form, used to evaluate nutritional status. |
| QoR-15 | 15-item Quality of Recovery scale, used to measure the quality of recovery post-surgery. |
| PROMIS-PF | Patient-Reported Outcomes Measurement Information System for physical functioning. |
| PROMIS-10 | Patient-Reported Outcomes Measurement Information System for quality of life. |
