# Supplementary material for: Feasibility of a Comprehensive eCoach to Support Patients Undergoing Colorectal Surgery: Longitudinal Observational Study
Source: JMIR Perioper Med. 2025 Feb 25;8:e67425. doi: 10.2196/67425 (PMC11897663; doi:10.2196/67425)
Supplement: Multimedia Appendix 3 [file periop_v8i1e67425_app3.docx]

**Multimedia** **Appendix 3. Compliance of the different items of the eCoach.**

| Item | Compliance % (median + IQR) | Response |
| --- | --- | --- |
| **Preoperative (+/- 30 days)** | 92 [87-95] |  |
| Physiotherapy visit, n=37 | 98 [90-100] | Yes: 33%  No: 67% |
| BORG cardio (6-20) n=36 | 92 [82-100] | 12.89 [12.31-14.24] |
| BORG strength (6-20) n=36 | 92 [82-100] | 12.89 [12.29-14.37] |
| Physical activity n=37 | 95 [85-100] | Yes: 86%  No: 14% |
| BORG physical activity (6-20) n=37 | 95 [85-100] | 12.06 [11.21-12.77] |
| Number of steps (counts) n=36 | 86 [72-93] | 5279 [3139-6493] |
| Protein intake, N=37 | 90 [84-97] | Yes: 97%  No: 3% |
| **Postoperative (7 days)** | 100 [100-100] |  |
| Monitoring recovery  (better, same, worse)  N=34 | 100 [100-100] | Better: 60%  Same: 35%  Worse: 5% |
| Temperature (degrees Celcius) n=30 | 100 [100-100] | 36.7 [36.5-37] |
| Defecation last 24 hours (yes/no) n=31 | 100 [100-100] | Yes: 96%  No: 4% |
| Vomiting past 24 hours (yes/no) n=31 | 100 [100-100] | Yes: 1%  No: 99% |
| Pain (1-10) n=31 | 100 [100-100] | 2.58 [1.13-4.35] |
| General pain medication (yes/no) n=31 | 100 [100-100] | Yes: 81%  No: 19% |
| Additional pain medication (yes/no) n=31 | 100 [100-100] | Yes: 20%  No: 80% |
| Wound healing properly (yes/no) | 100 [100-100] | Yes: 83%  No: 17% |
| Photo wound |  |  |
