# Supplementary material for: Feasibility of a Comprehensive eCoach to Support Patients Undergoing Colorectal Surgery: Longitudinal Observational Study
Source: JMIR Perioper Med. 2025 Feb 25;8:e67425. doi: 10.2196/67425 (PMC11897663; doi:10.2196/67425)
Supplement: Multimedia Appendix 4 [file periop_v8i1e67425_app4.docx]

**Multimedia** **Appendix 4. USE outcomes.**

| Question  N=26 | Median [IQR] | Disagree (1-3), n(%) | Neutral (4), n(%) | Agree (5-7), n(%) |
| --- | --- | --- | --- | --- |
| Usefulness |  |  |  |  |
| 1. It helps me be more effective. | 6 [5-7] |  | 5 (19.2) | 21 (80.8) |
| 1. It helps me be more productive. | 6 [5-7] |  | 4 (15.4) | 22 (84.6) |
| 1. It is useful. | 6 [5-7] |  | 2 (7.7) | 24 (92.3) |
| 1. It gives me more control over the activities in my life. | 5 [5-7] |  | 5 (19.2) | 21 (80.8) |
| 1. It makes the things I want to accomplish easier to get done. | 6 [5-6] |  | 6 (23.1) | 20 (76.9) |
| 1. It saves me time when I use it. | 5 [4-6] | 2 (7.7) | 9 (34.6) | 15 (57.7) |
| 1. It meets my needs. | 5 [4-6] | 3 (11.5) | 5 (19.2) | 18 (69.2) |
| 1. It does everything I would expect it to do. | 5 [4-6] | 5 (19.2) | 7 (26.9) | 14 (53.8) |
| Ease of use |  |  |  |  |
| 1. It is easy to use. | 6 [6-7] | 2 (7.7) | 1 (3.8) | 23 (88.5) |
| 1. It is simple to use. | 6 [6-7] | 2 (7.7) | 1 (3.8) | 23 (88.5) |
| 1. It is user friendly. | 6 [5-7] | 3 (11.5) | 1 (3.8) | 22 (84.6) |
| 1. It requires the fewest steps possible to accomplish what I want to do with it | 5 [4-7] | 2 (7.7) | 5 (19.2) | 19 (73.1) |
| 1. It is flexible | 5 [4-7] | 5 (19.2) | 3 (11.5) | 18 (69.2) |
| 1. Using it is effortless. | 6 [5-7] | 2 (7.7) | 3 (11.5) | 21 (80.8) |
| 1. I can use it without written instructions. | 6 [6-7] | 2 (7.7) | 2 (7.7) | 22 (84.6) |
| 1. I don't notice any inconsistencies as I use it. | 6 [4-7] | 3 (11.5) | 3 (11.5) | 20 (76.9) |
| 1. Both occasional and regular users would like it | 6 [4-7] | 1 (3.8) | 6 (23.1) | 19 (73.1) |
| 1. I can recover from mistakes quickly and easily. | 6 [4-6] | 2 (7.7) | 4 (15.4) | 20 (76.9) |
| 1. I can use it successfully every time. | 6 [5-7] | 3 (11.5) | 2 (7.7) | 21 (80.8) |
| Ease of learning |  |  |  |  |
| 1. I learned to use it quickly. | 6 [6-7] | 1 (3.8) |  | 25 (96.2) |
| 1. I easily remember how to use it. | 6 [5-7] |  | 2 (7.7) | 24 (92.3) |
| 1. It is easy to learn to use it. | 6 [6-7] | 1 (3.8) | 2 (7.7) | 23 (88.5) |
| 1. I quickly became skillful with it. | 6 [6-7] | 1 (3.8) | 1 (3.8) | 24 (92.3) |
| Satisfaction |  |  |  |  |
| 1. I am satisfied with it. | 6 [5-7] | 2 (7.7) | 4 (15.4) | 20 (76.9) |
| 1. I would recommend it to a friend. | 6 [5-7] | 1 (3.8) | 3 (11.5) | 22 (84.6) |
| 1. It is fun to use. | 6 [4-7] | 1 (3.8) | 6 (23.1) | 19 (73.1) |
| 1. It works the way I want it to work. | 6 [4-7] | 4 (15.4) | 4 (15.4) | 18 (69.2) |
| 1. It is wonderful. | 5 [4-7] | 4 (15.4) | 4 (15.4) | 18 (69.2) |
| 1. I feel I need to have it. | 5 [4-7] | 6 (23.1) | 5 (19.2) | 15 (57.7) |
| 1. It is pleasant to use. | 6 [4-6] | 2 (7.7) | 5 (19.2) | 19 (73.1) |
