# Supplementary material for: Feasibility of a Comprehensive eCoach to Support Patients Undergoing Colorectal Surgery: Longitudinal Observational Study
Source: JMIR Perioper Med. 2025 Feb 25;8:e67425. doi: 10.2196/67425 (PMC11897663; doi:10.2196/67425)
Supplement: Multimedia Appendix 5 [file periop_v8i1e67425_app5.docx]

**Multimedia** **Appendix 5. Quotes of patient experiences.**

| Positive experiences (n=46) |
| --- |
| General positive experiences (n=18) |
| 1. "I am very enthusiastic; I think this should be included in every care pathway." 2. "I found the pre-treatment phase very positive. I really felt myself getting fitter." 3. "I experienced the process itself as quite satisfactory." 4. "I found this method of guidance positive." 5. "The app is good." 6. "Overall, it's good." 7. "I am very positive about the app and would recommend it to everyone." 8. "The app is good." 9. "The app is great." 10. "I see no downsides to the app." 11. "I can recommend it to everyone. The app worked well for me." 12. "The entire prehabilitation process was excellent. The app was easy to use and I had a very positive experience." 13. "I felt well-prepared for the operation." 14. "The app was already very good in many areas, such as guidance on tapering medication." 15. "The app was a good tool and worked perfectly." 16. "The app works perfectly and is very effective. I was pleased with the program." 17. "I benefited greatly from it." 18. "I made grateful use of it." |
| Providing support and engagement (n=15) |
| 1. "I found the support from the app to be good. It kept me actively and positively engaged." 2. "The support in the app was effective." 3. "The app was supportive and motivating." 4. "I found the physiotherapy and protein intake very pleasant and helpful. The app was supportive in this, even after the operation." 5. "I feel positive about the activities I performed." 6. "It was well-organized for the exercises." 7. "Before the operation, the app was very helpful; the checks: have you done this, have you done that, are you experiencing this." 8. "And you are well reminded." 9. "The reminder for protein intake is nice." 10. "Very nice, the reminders." 11. "Also a helpful memory aid for everything you need to do, like taking protein, etc." 12. "I initially found it a bit challenging, but later appreciated having the guidance." 13. "I was very active, which made me feel ready and confident about the upcoming surgery." 14. "The app gave me the feeling of being connected. Instead of having no contact with the hospital until the surgery, I felt like I was still in touch." 15. "I really appreciated that there was still some follow-up care after the surgery." |
| Informative (n=7) |
| 1. "All information is conveniently in one place." 2. “Nice to have the information and actions every day. During hospitalization, the app helped the user by reminding the nurse to start Fraxiparine (which I hadn’t started yet), and it turned out I indeed needed it. Thanks to the app, I also already knew how to self-administer it.” 3. "Convenient to find information again.” 4. “It was very convenient that there was so much information available.” 5. “The app provides practical tools and guidance.” 6. “It was nice that the app provided information about reducing the medication.” 7. “Good information about proteins.” |
| Stimulating motivation and incentives (n=4) |
| 1. "The app was very motivating, especially for encouraging me to move more." 2. "It really motivated me." 3. "Especially for motivating movement, it made me feel fitter and stronger." 4. "It served as a real incentive." |
| Mental support (n=2) |
| 1. "It provides a mental boost; it makes me consciously focus on prehabilitation and offers distraction." 2. "Combining prehabilitation with the app is an excellent idea. It helps manage fears and intrusive thoughts. During times of uncertainty, it provides structure to my days. I’ve gained a lot mentally from it." |
| Continuous connection (n=2) |
| 1. "The app gave me the feeling of being connected. Instead of having no contact with the hospital until the surgery, I felt like I was still in touch." 2. "I really appreciated that there was still some follow-up care after the surgery." |
| **Proposed improvements (n=41)** |
| Limited usability (n=9) |
| 1. "There were unnecessary additional notifications, such as asking if I had a bowel movement. This led to a wrong workflow, and it continued to indicate that I hadn't had one." "I received a red notification for a pain score of 4, which led to a call. I felt that the call wasn't necessary at that time." "For new actions, I received a ping on my phone in the morning and an email in the evening. It would be better if the email were also a phone ping because I don’t always read emails in the evening." "External links in the actions could not be opened. Sometimes you can't click on the link, and sometimes when you can, it leads to an error message screen." 2. "At the start, the information was confusing, with language issues and outdated phone numbers of the hospital. This has now been corrected." 3. "Initially, there were language problems with German, English, and Dutch mixed together. This has improved, though." 4. "There were language issues at the beginning, but they have been well resolved." 5. "There were problems with the transition to the discharge protocol.” 6. “There are still some technical issues here and there." 7. "There are still too many issues with the app at the moment. I see these as initial difficulties, but the app should be able to address these better, in my opinion." 8. "The phone numbers for daytime and nighttime calls didn’t work or were inefficient." 9. "The video about the surgery that was included couldn’t be viewed in the app. I had to go to the hospital’s website to watch it." 10. “The app was too complicated, so I filled it with my informal caregiver" 11. If you’re not tech-savvy, the app can be confusing. It sometimes isn't clear what you have already done and what you still need to do. The same questions are asked under other components of the app, which can be confusing." |
| Rigidity of the app (n=14) |
| 1. "The interaction regarding the prehabilitation process was not optimal." “It's unfortunate that you can’t ask questions in between (noreply).” 2. "The app assumes an average person and doesn’t account for personal differences, which makes it difficult for me to provide an accurate average for myself." "It’s annoying that you can't enter actions the next day if you forget. I had a few 'top scores' for steps and wanted to record them, but I forgot and couldn’t register them from the previous day. I found this very disappointing." 3. "Many features of the app were less relevant for me due to my spinal cord injury, making them less useful. I was also hospitalized for 2 weeks after the surgery, so I couldn’t use the app during that time." 4. "I use a wheelchair, and I initially received questions about the number of steps. This feature was turned off by the nurse." 5. "I found it disappointing that I couldn’t specify why certain things weren’t working. For example, I had anemia, and sometimes I couldn’t complete the daily exercise before surgery. I couldn’t explain this in the app." 6. "Sometimes I didn’t meet my step goals due to rainy weather. It was frustrating that I couldn’t indicate the reason for not reaching my step count." 7. "For pre-surgery movement, it would be helpful to have a comment field where you could note things like extra household work done or additional steps taken." "It's frustrating that forgotten actions disappear and can no longer be entered." 8. "I found it difficult that the answer options were only yes/no, with no option to add comments." 9. "There was little variation in the pain questions. I couldn’t specify if the pain was in a different location." 10. "I would like to see more options for providing explanations." 11. "The next morning, I couldn't enter the action in the app anymore. The actions were still listed, but the ball didn’t turn blue, which I think means that Luscii didn’t save it. The actions remained open." 12. "You can only enter actions on the same day; it would be better if they remained available for a bit longer." 13. "It’s inconvenient that if you forget to enter an action (like steps in the evening), you can't enter it the next day." 14. "The app repeatedly asked if I wanted to send a photo, which made me think I needed to take a picture of my wound. I wasn’t worried, but I did send the photo." |
| Problems with the pedometer (n=6) |
| 1. "My phone doesn't integrate with the app for step tracking. It has to be entered manually, which is disappointing." 2. "The step counter doesn’t match my own step counter (the iPhone step counter is linked to the app); it shows many fewer steps, which is frustrating." 3. "I didn’t have a step counter, so I didn’t record steps." 4. "He didn’t have a step counter, so the steps were entered based on estimate." 5. "Technically, the step count is often a day behind." 6. "The step count was not always accurate; the app didn’t always sync properly with the phone." |
| Length of postoperative monitoring was unclear or insufficient (n=6) |
| 1. "It's unfortunate that the app stops so quickly after surgery. I would have preferred a gradual phase-out and to keep receiving messages or advice (like tips and tricks on lifestyle, rebuilding fitness, home exercises) for a bit longer post-surgery." 2. "I found it disappointing that there was no guidance on protein intake after the surgery. I kept taking the proteins, but it’s a shame the support stopped." 3. "I was disappointed when the 'How are you?' questions stopped after surgery. In my case, with some wounds still healing, I would have liked to continue with them." 4. "I wish the app had a section for unforeseen issues after discharge with some information on what to do." 5. "Before surgery, there was a full program with physiotherapy, but after surgery, it suddenly felt very empty. I would have liked continued guidance from a physiotherapist afterward." 6. "After the surgery, I felt fine and thought, 'With all these questions, I’m just going to delete the app.' I also couldn’t find any information about how or when the app would be discontinued." |
| Missed features in the app (n=3) |
| 1. "It's a pity that activities like cycling can't be tracked." 2. "It would be helpful to add a feature in the app for thrombosis injections, so you receive a reminder asking if you’ve taken your thrombosis medication." 3. "There’s also no feature for tracking weight, which would be useful since you’re not supposed to lose weight. It would be a good tool to monitor this." |
| Engagement difficulties and mental burden (n=3) |
| 1. "I didn’t always feel motivated to fill it out." 2. "It wasn't really special; I didn’t get much out of it. There was too much going on." 3. "I found using the app very intensive. It kept me engaged every day, which I found mentally exhausting." |
