# Supplementary material for: Feasibility of a Comprehensive eCoach to Support Patients Undergoing Colorectal Surgery: Longitudinal Observational Study
Source: JMIR Perioper Med. 2025 Feb 25;8:e67425. doi: 10.2196/67425 (PMC11897663; doi:10.2196/67425)
Supplement: Multimedia Appendix 6 [file periop_v8i1e67425_app6.docx]

**Multimedia** **Appendix 6. App-induced nursing activities.**

| Item | Number of patients \| Number of patients with alert \| percentage | Alerts \| Alerts automatic processed \| percentage | Action |
| --- | --- | --- | --- |
| **Preoperative (+/- 30 days)** |  |  |  |
| Physiotherapy visit | 37 \| 23 \|62% | 186 \|186 \|100% | - |
| BORG cardio | 36 \| 24 \| 67% | 47 \| 47 \|100% | - |
| BORG strength | 36 \| 24 \| 67% | 47 \| 47 \|100% | - |
| Physical activity | 37 \| 24 \| 65% | 79 \| 79 \| 100% | - |
| BORG physical activity | 37 \| 24 \| 65% | 79 \| 79 \| 100% |  |
| Number of steps | 36 \| 35 \| 97% | 1092 \| 1092 \| 100% |  |
| Protein intake | 37 \| 30 \| 81% | 222 \| 220 \| 99% | Message: 2 (1%) |
| Preoperative sum | 256 \| 184 \| 72% | 1752 \| 1750 \| 99.9% |  |
| **Postoperative (7 days)** |  |  |  |
| Monitoring recovery | 34 \| 13 \| 38% | 44 \|19 \| 43% | No action: 16 (41%)  Message: 7 (18%)  Phone call: 2 (5%) |
| Temperature | 30 \| 4 \| 13% | 4 \| 3 \| 75% | No action: 1 (25%) |
| Defecation | 31 \| 4 \| 13% | 4 \| 3 \| 75% | No action: 1 (25%) |
| Vomiting | 31 \| 5 \| 16% | 5 \| 3 \| 60% | No action: 1 (20%)  Phone call: 1 (20%) |
| Pain | 31 \| 10 \| 32% | 19 \| 7 \| 37% | No action: 6 (13%)  Message: 1 (7%)  Phone call: 5 (33%) |
| General pain medication | 31 \| 9 \| 29% | 15 \| 13 \| 87% | No action: 1 (8.5%)  Phone call: 1 (8.5%) |
| Additional pain medication | 31 \| 9 \| 29% | 10 \| 5 \|50% | No action: 3 (30%)  Message: 1 (10%)  Phone call: 1 (10%) |
| Wound healing properly | 31 \| 10 \| 32% | 18 \| 1 \| 6% | No action: 3 (17%)  Message: 8 (44%)  Phone call: 6 (33%) |
| Photo wound | 31 \| 6 \| 19% | 7 \| 0 \| 0% | Message: 1 (14%)  Phone call: 5 (72%)  Sent photo to nurse: 1 (14%) |
| Postoperative sum | 281 \| 70 \| 25% | 126 \| 54 \| 43% |  |
